# Planting *Jatropha curcas* on Constrained Land: Emission and Effects from Land Use Change

**DOI:** 10.1100/2012/405084

**Published:** 2012-04-01

**Authors:** M. S. Firdaus, M. H. A. Husni

**Affiliations:** Department of Land Management, Faculty of Agriculture, Universiti Putra Malaysia, Selangor, 43400 Serdang, Malaysia

## Abstract

A study was carried out to assess carbon emission and carbon loss caused from land use change (LUC) of converting a wasteland into a *Jatropha curcas* plantation. The study was conducted for 12 months at a newly established *Jatropha curcas* plantation in Port Dickson, Malaysia. Assessments of soil carbon dioxide (CO_2_) flux, changes of soil total carbon and plant biomass loss and growth were made on the wasteland and on the established plantation to determine the effects of land preparation (i.e., tilling) and removal of the wasteland's native vegetation. Overall soil CO_2_ flux showed no significant difference (*P* < 0.05) between the two plots while no significant changes (*P* < 0.05) on soil total carbon at both plots were detected. It took 1.5 years for the growth of *Jatropha curcas* to recover the biomass carbon stock lost during land conversion. As far as the present study is concerned, converting wasteland to *Jatropha curcas* showed no adverse effects on the loss of carbon from soil and biomass and did not exacerbate soil respiration.

## 1. Introduction

The onset of the Industrial Revolution has seen an increase of 110 ppmv carbon dioxide (CO_2_) in our atmosphere resulting in the current atmospheric CO_2_ concentration to be more than 390 ppmv [1]. Burning of fossil fuel had been the largest source of CO_2_ emission followed by agriculture and land use change where, in 2005, the two activities made up 48% and 31% of the total global CO_2_ emission, respectively [2].

Nonconventional fuel such as biodiesel from *Jatropha curcas* had been largely explored to be the alternative to fossil fuel in reducing CO_2_ emission. The advantage of using *Jatropha curcas* compared to other biofuel crops is that it is nonedible; therefore, it does not create a conflict of utilizing food for fuel. It is also the third highest oil producing crop in terms of oil yield per hectare after oil palm and coconut where it could produce 2.236 L oil ha^−1^ under optimum field condition [3]. Being a perennial crop, it does not require frequent removal of biomass and soil tillage and can continue producing seeds of which the oil is extracted from before needing to be replanted after 25 years. Apart from that, it is a hardy plant which requires minimum irrigation and fertilization making it possible to be cultivated on marginal soil and on dry parts of the world [4].

Biodiesel from *Jatropha curcas* was claimed to emit less greenhouse gases in particular CO_2_ compared to diesel from fossil fuel. A number of life cycle analysis (LCA) studies were carried out on the production and combustion of biodiesel from *Jatropha curcas *and most studies concluded that there is a reduction in emission of greenhouse gases when compared to conventional diesel. Life cycle analysis studies by Kritana and Gheewala [5], Dehue and Hettinga [6], and Ndong et al. [7] for instance showed 77%, 68%, and 72% of greenhouse gas reduction, respectively.

Nevertheless, most of the LCA studies that are currently available had not put much emphasis on the emissions from land use change (LUC) of establishing a *Jatropha curcas *plantation despite of LUC being the second largest contributor of the total global greenhouse gas emission. None of the reviewed LCAs had used actual primary data on the effects on land use change particularly on changes of carbon stock and CO_2_ emission from soil. Most of the LCA studies either used standard factors published by the IPCC [8], secondary data from other published studies, or simply made assumptions without concrete scientific basis. Past LCA studies had emphasized on CO_2_ emission caused by energy consumption during production and transportation of *Jatropha curcas* biodiesel and emission from its combustion with less focus being placed on gathering data on the effects of LUC.

This study was, therefore, carried out to make field assessment on the emissions or perhaps sequestration that arises as a result of converting a wasteland to a *Jatropha curcas* plantation. Subsequently, assessment from this study would be able to fill the gap left out by most of the LCA studies on emissions from LUC. The specific objectives of this study were to (i) quantify carbon stock loss during land conversion and regeneration of new carbon stock through *Jatropha curcas* biomass production, (ii) to compare the soil CO_2_ fluxes between a *Jatropha curcas* plantation and a wasteland, and (iii) to quantify changes in soil carbon at both land use types.

## 2. Materials and Methods

### 2.1. Site Description

The study was conducted at Tanah Merah Estate at Port Dickson, Negeri Sembilan, Malaysia (2° 39′ 32.51′′N, 101° 47′ 07.55′′E). The estate is an oil palm plantation with a total area of slightly less than 500 ha. Approximately, 11 km of 550 kV electricity pylon runs through the estate covering an area of 45.68 ha of which could not be planted with oil palm due to height restriction. The area under the pylon had since turned into wasteland which is defined as a barren uncultivated land covered with wild shrubs. The wasteland underneath the pylon was predominantly covered by *Lantana camara*,* Paspalum sp.*,* Digitaria sp.*,* Axonopus sp., *and *Argyreia sp.*

In 2008, the company that owned the oil palm plantation had decided to convert the wasteland into a *Jatropha curcas* plantation since its height which was kept at 2 m would not obstruct the cables of the pylon. Land preparation activities which included removing the native vegetation using a bulldozer and two repetitions of soil plowing was carried out in November 2008. The first planting of *Jatropha curcas* saplings began in January 2009.

Average monthly temperature at the site was 30°C with an annual rainfall of 2200 mm [9]. The soil at the area is a Typic Paleudult with a sandy clay to sandy clay loam texture [10].

### 2.2. Experimental Setup

The study began in August 2009 until July 2010. Two study plots were established at the site. The plot that was planted with *Jatropha curcas* was designated as plot P while the wasteland was designated as plot S. The size of both plots is approximately one hectare locating next to each other with both plots having an approximately 7% slope.

At plot P, five 3 × 3 m quadrats were randomly placed. Each quadrat in plot P comprised of four *Jatropha curcas* trees. Soil sampling and soil flux measurements were conducted within the five quadrats at plot P. Stem diameter of each tree in every quadrat (*n* = 20) was measured monthly at plot P.

At plot S, five 3 × 3 m quadrats were also randomly placed. Within these quadrats, however, only soil CO_2_ flux measurements were made. Another five 3 × 3 m quadrats were randomly placed at plot S for the removal of native vegetation at the wasteland during the initial stage of the study.

### 2.3. Biomass Determination

#### 2.3.1. Biomass at Plot P

Destructive sampling of *Jatropha curcas* trees of different stem diameter and ages was made to determine the allometry relationship between increments of stem diameter with increments of its biomass dry weight in accordance with the methods of Basuki et al. [11]. The destructive sampling was carried out in a *Jatropha curcas *plot at Universiti Putra Malaysia, Serdang, Malaysia. It was assumed that *Jatropha curcas* trees at Universiti Putra Malaysia would be representative to the trees at Port Dickson as soil at both sites are of the same soil series, and both sites were also planted with the same local “Sengkarang” accession.

Fifteen trees were randomly chosen and were sawn off as close as possible to the ground. The aboveground sections of the trees were then separated to its main stems, branches, and leaves. All the parts were directly weighed in the field for the determination of their fresh weight. The belowground sections of the trees (i.e., roots) were excavated using a backhoe, and soil particles attached to the roots were washed with water before the roots were weighed for determination of fresh weight.

Three replications of subsamples were made on the root, stem, and branch for moisture content determination where the samples were oven dried at 60°C to a constant weight. Dry matter of the whole tree was then calculated by subtracting the moisture content from the fresh weight of the felled trees.

Equations based on the allometry relationship were then generated by plotting the natural log transformed aboveground and belowground biomass dry weight against its respective transformed stem diameters. The plots were then fitted to a linear regression, and the linear models generated were used to estimate the above and belowground biomass dry weight

(1) ln⁡⁡(BDM)=  c+aln⁡⁡(SD),

where BDM is biomass dry matter in kg, SD is stem diameter in cm, *c* is the intercept, and *a* is the slope coefficient of the regression.

At plot P, stem diameter of 20 *Jatropha curcas* trees was measured every month using a digital caliper (Mitutoyo, Japan). Two measurements were made on each tree running latitudinal and longitudinally, and the average of the two measurements was recorded as its stem diameter. The stem diameter measurements were then used to calculate the current month's above and belowground biomass by using the allometric equations generated. The average biomass for the sampled 20 trees was then extrapolated to a hectare scale based on the assumed planting density of 1600 trees ha^−1^ [12].

#### 2.3.2. Biomass at Plot S

Vegetations within the five quadrats randomly placed at plot S was clipped and were directly weighed to determine its fresh weight. Three replications of subsamples from the vegetation at each quadrat were made and oven dried at 60°C to a constant weight for moisture content determination. Biomass dry weight was then estimated by subtracting the fresh weight of the removed biomass by its moisture content. Estimated biomass from the sampled area of 45 m^2^ was then extrapolated to an area of one hectare.

#### 2.3.3. Quantification of Litterfall Production

Litterfall was collected monthly to quantify biomass production through litterfall production. Five litter traps were constructed under the canopy of five randomly selected *Jatropha curcas* trees at plot P. The litter traps was made of nylon fishing net covering an area of 4 m^2^ under each canopy. The trapped litterfall was removed monthly and transferred into paper bags. The litterfall were then oven dried at 60°C to a constant weight, and the final weights were recorded as its dry matter.

### 2.4. Determination of Carbon in Biomass

Subsamples of the different parts from the destructive harvesting of *Jatropha curcas *trees and monthly collected litterfall were used in the determination of carbon in biomass. The dried sampled parts of the *Jatropha curcas* trees were sheared to smaller pieces using secateurs before they were ground to sizes of less than 5 mm using a kitchen mill. Litterfall was ground to sizes of less than 2 mm using a cutting mill (IKA, Germany). All samples were then analyzed for carbon content by the combustion method using the CR-412 carbon analyzer (LECO, Mich, USA).

The percentages of carbon in the different parts of the biomass were then used to estimate the mass of carbon in biomass based on biomass dry weight. The estimated biomass per hectare was then converted into mass of carbon in biomass per hectare.

### 2.5. Soil Total Carbon

Soil sampling was carried out to determine the monthly changes of total soil carbon. One soil sample was sampled from each quadrat at both plot P and plot S at a depth of 0 to 20 cm from the soil surface using a soil auger every month. The soil samples were then air dried ground and sieved through a 2 mm sieve before being sent for total carbon analysis also by using the CR-412 carbon analyzer (LECO, Mich, USA).

### 2.6. Soil CO_2_ Flux

Soil CO_2_ flux was measured to determine the amount of carbon lost from soil as CO_2_ to the atmosphere at both plots. Measurements were made by using the LI-8100 automatic soil CO_2_ flux system (LI-COR Biosciences, Neb, USA). Flux measurements were conducted at both plots where five measurements were made within the assigned quadrats of each plot.

Prior to making flux measurement, a soil collar made of a 10 × 10 cm (d × h) PVC pipe was inserted into the soil for at least an hour to allow the disrupted soil and sheared fine roots to stabilize. Soil CO_2_ flux measurements were made between 1000 and 1200 hrs where daily soil CO_2_ flux was at its highest rate [13].

The flux rate was calculated by fitting the changes in concentration of CO_2_ within the chamber of the flux system to either an exponential or a linear regression which was done by the system's software and given in units of *μ*mol CO_2_ m^−2^ s^−1^.

### 2.7. Statistical Analyses

Comparison of soil CO_2_ fluxes between plots P and S was made by using a one-way *t*-test. Linear regression analysis was conducted to determine the relationship between two parameters of interest (e.g., biomass versus stem diameter). Analysis of variance and mean separation by Tukey's test were used for the determination of significant differences among means. All data sets were analyzed for outliers by using the Grubb's test [14].

## 3. Results

### 3.1. Dry Matter Production and Sequestered Carbon in Biomass

The mean stem diameter, mean dry weight, and mean moisture content of the aboveground and belowground sections of the 15 *Jatropha curcas* sampled are presented in Table 1 categorized by age.

**Figure 1:**
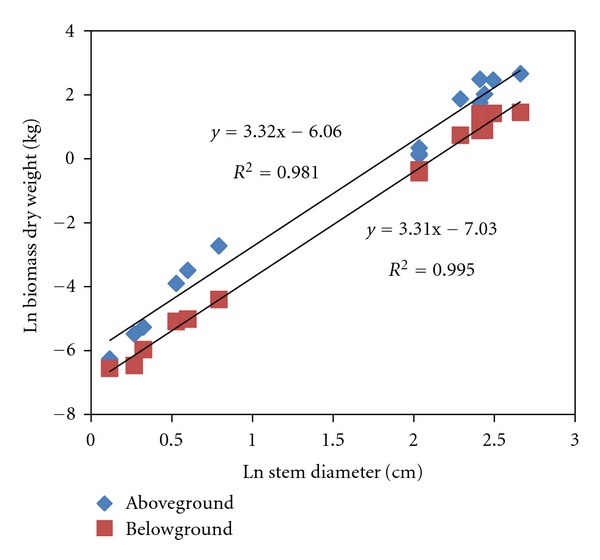
Linear regression of the stem diameter and biomass dry weight of the aboveground and belowground sections of *Jatropha curcas*.

The natural log transformed biomass dry weight of the aboveground and the belowground sections of the trees plotted against its respective log transformed stem diameter are shown in Figure 1. The two plots were then regressed to a linear model.

The two allometric equations generated based on the linear regressions of the aboveground and belowground sections were

(2) $$\begin{array}{cc} \ln\left(\text{AGDW}\right) & =3.32\left(\ln\;\text{SD}\right)-6.06, \end{array}$$

(3) $$\begin{array}{cc} \ln\left(\text{BGDW}\right) & =3.11\left(\ln\;\text{SD}\right)-7.03 \end{array},$$

where AGDW and BGDW are the aboveground and belowground dry weights in kg, respectively, while SD is the stem diameter in cm.

The percentage of carbon in leaves and the aboveground and belowground sections of the composite biomass samples are presented in Table 2.

From the generated allometric equations (1) and (2), estimation of monthly biomass dry weight was made based on measured stem diameter where subsequently mass on carbon in biomass was calculated based of carbon content in biomass (Table 3). Litterfall production and mass of carbon in litterfall was also listed in Table 3. All figures had been extrapolated to a hectare scale.

Total biomass dry weight of the vegetation removed from plot S from five quadrats was 11.45 kg for an area of 45 m^2^ (Table 4). By extrapolating it to a hectare scale, the mean biomass dry matter at plot S was estimated to be 2.54 Mg ha^−1^. An assumption of 1 : 2.5 root to shoot ratio typical for tropical shrubs and undergrowth [15] was used to calculate then the belowground biomass of the removed vegetation, and it was estimated to be 1.02 Mg ha^−1^. Adding up the above- and belowground sections yields 3.56 Mg of biomass dry matter per hectare.

### 3.2. Soil Carbon

Table 5 shows soil carbon content at both plots sampled from the five quadrats at each plot expressed in percentage of carbon. Both plots showed no significant differences (*P* < 0.05) between months by ANOVA within each plot.

### 3.3. Soil CO_2_ Flux

**Figure 2:**
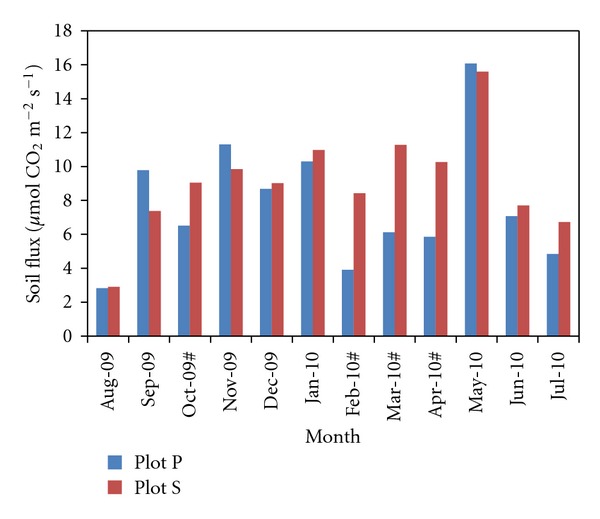
Mean soil flux at plot P and plot S. Months with a # sign indicates that there is a significant difference by *t*-test (*P* > 0.05).

Soil CO_2_ fluxes from plot P and plot S are presented in Figure 2. Mean soil CO_2_ flux at plot P ranged from 2.83 *μ*mol m^−2^ s^−1^ to 16.08 *μ*mol m^−2^ s^−1^. Meanwhile at plot S, soil CO_2_ flux ranged from 2.91 *μ*mol m^−2^ s^−1^ to 15.59 *μ*mol m^−2^ s^−1^. The minimum and maximum fluxes at both plots were recorded at August 2009 and May 2010, respectively.

During most months, monthly soil CO_2_ flux at plot P was not significantly different (*P* < 0.05) by *t*-test from flux at plot S with the exception during October, February, March and April where fluxes at plot S were significantly higher (*P* > 0.05) than at plot P.

## 4. Discussion

### 4.1. Biomass Production and Carbon in Biomass

Carbon content in aboveground and belowground biomass of 45.60% and 44.68%, respectively, was found to be lower than the carbon content observed by CMSCRI [16] of 50.9% and 51.5% for the aboveground and belowground biomass, respectively, of a one-year-old *Jatropha curcas* tree. Mean dry weight of *Jatropha curcas* trees of the present study, however, was observed to be higher than that of CMSCRI where mean biomass dry weight of the present study is 1.64 Mg ha^−1^ compared to 0.49 Mg ha^−1^ of CMSCRI despite being the same age. Recorded biomass dry weight of the present study at 46 months was also found to be higher than that of CSMCRI [15] at 42 months where mean total biomass dry weight of the present study is 10.89 kg tree^−1^ compared to 5.5 kg tree^−1^ of CSMCRI albeit a four months difference between the trees.

The discrepancies in biomass carbon content of the two studies might be due to the different lignin content in the biomass from the two studies [17]. Lignin was not analyzed in the two studies but an analysis by Vaithanomsat and Apiwatanapiwat [18] found lignin content in *Jatropha curcas* stem to be 24.11%. The discrepancies of the different biomass dry weight of the two studies might possibly be due to the agronomic practices of the two plantations and site characteristics of the two studies.

Nevertheless, based on the biomass carbon content and dry weight, an estimated 0.74 Mg C ha^−1^ was sequestered in biomass of a one-year-old *Jatropha curcas* from the present study as opposed to only 0.25 Mg C ha^−1^ of CMSCRI [15]. Meanwhile, carbon sequestration in three-years-old *Jatropha curcas* of the present study, and that of CMSCRI was 7.84 and 4.40 Mg C ha^−1^, respectively. The large differences between the two studies might suggest that quantification of biomass production of *Jatropha curcas* have to be made according to specific sites.

Total litterfall production of 1.29 Mg ha^−1^ of the present study somewhat agrees with the result of Abugre et al. [19] who found that litterfall production of *Jatropha curcas* planted at planting distances of 1 m × 1 m, 2 m × 1 m, and 3 m × 1 m to be 2.27, 1.10 and 0.79 Mg ha^−1^, respectively. According to the same study by Abugre et al. [19], after 120 days of decomposition, between 2.45 and 34.6% carbon is still left from *Jatropha curcas* litterfall. The large difference in the decomposition rate is due to the difference in sunlight exposure on the litterfall [20].

The amount of carbon stock that was removed when converting the wasteland into *Jatropha curcas* was estimated to be 1.78 Mg carbon ha^−1^ assuming the carbon content of the shrubs at plot S to be 50%. This value is lower than estimated value of 3.10 Mg C ha^−1^ when converting tropical grassland to *Jatropha curcas* [6].

Based on *Jatropha curcas* biomass growth of the present study, it only took 1.5 years for *Jatropha curcas* to recover back the initial carbon stock that was lost during the land clearing process. Carbon stock of plot P at 18 months after planting was 1.86 Mg ha^−1^. As far as the time required for replenishing lost carbon stock from land conversion is concerned, result of the present study is faster than what was concluded by Fargione et al. [21] and Romijn [22] that estimated 20 to 30 years to recover lost carbon from biomass as a result of LUC for biofuel production.

### 4.2. Soil CO_2_ Flux

Soil fluxes at the two sites showed no significant differences throughout the observation period apart from during October 2009, February, March, and April 2010 where soil flux at plot S was significantly higher than that at plot P. This indicated that soil preparation activities (i.e., the removal of native vegetation and soil tillage) did not cause an increase in CO_2_ emission from the soil which contradicts with other previous observations [23–25]. As a matter of fact, four out of the twelve months of observation showed that CO_2_ fluxes at plot S were higher than those at plot P.

No changes in total soil carbon content were detected at both plots during the observation period. This again showed that land preparation activity did not have much influence on soil carbon. The results contradicted with the report of Romijn [22] who found a net loss of soil organic carbon as much as 32 Mg ha^−1^ on a *Jatropha curcas* plantation converted from a virgin Miombo Woodland. The LCA study by Dehue and Hettinga [6] on the other hand assumed that no carbon buildup occurs in the soil even after 20 years of planting.

## 5. Conclusion

No significant losses were detected at least during the first year of cultivation on soil carbon and by means of soil CO_2_ fluxes. Within less than one and a half year, the initial carbon stock that was removed during land preparation was recovered back by the growth of *Jatropha curcas* trees. It could, therefore, be concluded that converting a wasteland into a *Jatropha curcas* plantation does not show any degrading effects on LUC at least in the case of the present study.

## Figures and Tables

**Table 1:** Mean ± standard error of stem diameter, dry weight of the aboveground and belowground section of *Jatropha curcas*, and its moisture content at different ages.

Age (month)	Stem diameter (cm)	DWAG (kg)	Moisture (%)	DWBG (kg)	Moisture (%)
46	14.6 ± 1.0	7.72 ± 0.25	65.8 ± 0.5	3.17 ± 0.07	67.1 ± 0.2
32	12.5 ± 0.9	5.88 ± 0.58	68.4 ± 0.8	2.27 ± 0.05	64.7*
24	9.3 ± 0.4	2.23 ± 0.21	70.7 ± 0.1	1.03 ± 0.37	67.2 ± 2.1
10	7.6 ± 0.0	0.45 ± 0.02	73.0 ± 0.6	0.47 ± 0.02	58.8*
<6	1.6 ± 0.2	0.02 ± 0.01	n.a	0.01 ± 0.00	n.a

DWAG: dry weight aboveground.

DWBG: dry weight belowground.

n.a: not applicable.

*: no replications were made for moisture content determination.

**Table 2:** Mean carbon content of the different parts of *Jatropha curcas*.

Plant part	Carbon content (%)
Aboveground	45.60
Belowground	44.86
Leaf	46.46

**Table 3:** Estimated monthly dry weight of biomass and litterfall of *Jatropha curcas* and mass of carbon stored in each respective part (mean ± standard error).

Month	Year	Biomass dry weight	Carbon in biomass	Litterfall dry weight	Carbon in litterfall
		(Mg ha^−1^)		(Mg ha^−1^)	
August	2009	0.60 ± 0.38	0.27 ± 0.17	0.05 ± 0.02	0.03 ± 0.01
September	2009	0.83 ± 0.49	0.38 ± 0.22	0.03 ± 0.02	0.01 ± 0.01
October	2009	1.02 ± 0.48	0.46 ± 0.22	0.08 ± 0.05	0.04 ± 0.03
November	2009	1.04 ± 0.50	0.47 ± 0.23	0.11 ± 0.08	0.05 ± 0.04
December	2009	1.44 ± 0.69	0.65 ± 0.31	0.18 ± 0.09	0.08 ± 0.04
January	2010	1.64 ± 0.76	0.74 ± 0.34	0.08 ± 0.04	0.04 ± 0.02
February	2010	2.06 ± 0.98	0.93 ± 0.44	0.11 ± 0.03	0.05 ± 0.02
March	2010	2.42 ± 1.07	1.10 ± 0.48	0.05 ± 0.05	0.02 ± 0.02
April	2010	2.99 ± 1.53	1.18 ± 0.58	0.12 ± 0.09	0.06 ± 0.04
May	2010	3.69 ± 1.88	1.46 ± 0.73	0.12 ± 0.04	0.06 ± 0.02
June	2010	4.09 ± 1.82	1.86 ± 0.82	0.16 ± 0.07	0.08 ± 0.03
July	2010	4.68 ± 1.51	2.13 ± 0.68	0.22 ± 0.07	0.10 ± 0.03

Cumulated Total		4.08	1.86	1.29	0.60

**Table 4:** Biomass dry weight of aboveground vegetation removed at plot S from five replications of 9 m^2^ quadrats.

Quadrat (3 × 3 m)	Biomass dry weight (kg)
1	2.30
2	1.05
3	3.85
4	1.45
5	2.80

Total	11.45

**Table 5:** Changes of soil carbon content at plot P and plot S.

	2009					2010						

Month	Aug	Sep	Oct	Nov	Dec	Jan	Feb	Mar	Apr	May	Jun	Jul
					Carbon (%)							
Plot S	2.12	2.09	1.76	2.59	1.80	2.35	2.67	1.83	2.13	2.05	1.87	1.81
Plot P	1.63	2.20	1.94	1.16	1.35	1.42	1.62	1.56	1.74	1.80	1.66	1.75
